# Understanding Neuromuscular System Plasticity to Improve Motor Function in Health, Disease, and Injury

**DOI:** 10.1155/2017/2425180

**Published:** 2017-03-02

**Authors:** Guang H. Yue, Brian C. Clark, Sheng Li, David E. Vaillancourt

**Affiliations:** ^1^Kessler Foundation and Rutgers University, West Orange, NJ, USA; ^2^Ohio Musculoskeletal and Neurological Institute (OMNI), Ohio University, Athens, OH, USA; ^3^Department of Physical Medicine and Rehabilitation, University of Texas Health Sciences Center at Houston, Houston, TX, USA; ^4^Department of Applied Physiology and Kinesiology, University of Florida, Gainesville, FL, USA

Disease or injury of motor system components often leads to motor dysfunction, and the success of medical intervention, motor skill learning, exercise, or sports training is linked to plasticity in the neuromuscular system at both the central and peripheral levels. However, the neuroplasticity mechanisms underlying the therapeutic efficacy of motor function rehabilitation, exercise training, or motor learning on the neuromuscular system are not well understood. The lack of knowledge on plasticity at various levels of the central and peripheral motor systems, including the muscle, limits a good understanding of neural mechanisms provoking movement disorders and their recovery and hinders development of targeted therapies for effective treatment. In addition, understanding neuroplasticity linked to motor learning may help identify practice strategies to maximize the progressive plasticity and relearning of motor skills. In the field of sports training, profound insights into plasticity of the neuromuscular system may help develop unique training regimes to aid athletes reach their maximal potential and at the same time prevent injury. The special issue in this journal solicited high quality, original research articles as well as review articles focused on plasticity of central and/or peripheral motor systems, including the muscular system as a result of motor system disease, injury, and rehabilitation; motor skill learning; and exercise or sports training. Approximately half of the articles in this issue center on the development of new methodologies to evaluate neuroplasticity. More specifically, Q. She and colleagues report a novel method to identify classes of electroencephalography (EEG) signals during different motor imagery tasks, while T. Nguyen and colleagues report a novel multimodal EEG/MRI integration method to achieve high spatiotemporal accuracy. M. Chen and colleagues report that a novel progressive FastICA peel-off (PFP) framework technique for decomposing high density surface EMG signals into different motor units yields a high degree of agreement with the more common convolution kernel compensation (CKC) method, and they suggest that combination of the two methods may have the potential to further increase the decomposition yield. D. Kraus and A. Gharabaghi describe a novel projection, interpolation, and coregistration technique, which considers the individual gyral anatomy, to acquire TMS motor maps that demonstrated long-term, high test-retest reliability. Lastly, H. Peters and colleagues utilized transcranial magnetic stimulation to elicit motor evoked potentials (MEPs) from tibialis anterior and soleus muscles of thirty-five chronic stroke patients with lower extremity hemiparesis and observed that a prolonged MEP latency was associated with reduced lower extremity physical function. As such, they suggest that MEP latency could serve as a stroke-related biomarker.

The other articles center on interventional strategies to promote neuromuscular system plasticity. Here, S. Madhavan and colleagues report that a single session of high-intensity interval treadmill exercise suppresses corticomotor excitability in the paretic muscles of some (~65%) chronic stroke survivors and that when this exercise is preceded by transcranial direct current stimulation in combination with a skill acquisition task, the asymmetry of between-hemisphere corticomotor excitability is reduced. M. Wu and colleagues report data suggesting that improved weight shifting induced by robotically applied pelvis assistance force during stance may facilitate stepping in children with cerebral palsy (CP) and that applying large leg swing assistance force during treadmill training may reduce the active participation of children with CP. Lastly, F. Steinberg and colleagues report data suggesting that mirror visual feedback facilitates intermanual transfer effects in sport, but only for participants that had experience with the movements being performed. As such, they introduce and discuss the role of skill level and task complexity in the field of mirror visual feedback.

Collectively, we hope this series of articles will, in the long term, help to advance our understanding of neural mechanisms (plasticity) of motor function disorders and will facilitate the development of targeted therapies to maximize the plasticity for effective treatment, leading to fast recovery of motor function.

*Guang H. Yue* *Brian C. Clark* *Sheng Li* *David E. Vaillancourt*

